# Evaluation of haematological, genotoxic, cytotoxic and ATR-FTIR alterations in blood cells of fish *Channa punctatus* after acute exposure of aniline

**DOI:** 10.1038/s41598-023-48151-z

**Published:** 2023-11-25

**Authors:** Geetika Sharma, Pooja Chadha

**Affiliations:** Cytogenetics Lab, Department of Zoology, Guru Nanak Dev University, Amritsar, 143005 India

**Keywords:** Zoology, Environmental sciences

## Abstract

Aniline (C_6_H_5_NH_2_) an important intermediate in the organic and fine chemical industry, is ubiquitously used worldwide. It is one of the important building block for manufacturing of 4,4-methylene diphenyl diisocyanate (MDI), accelerators in rubber processing, dyes, tattoo inks, photographic chemicals, antioxidants, corrosion inhibitors, pharmaceuticals and antiseptics. The current study evaluated 96 h LC_50_ of aniline and based on this, two sublethal concentrations (4.19 mg/l and 8.39 mg/l) were selected for acute exposure studies in freshwater food fish *Channa punctatus*. Erythrocytes of fish are nucleated hence they play an important role in physiology, immune system, protein signalling and haemostatic condition along with respiration. Blood samples were collected after 24, 48, 72, and 96 h of exposure to study haematological, cytotoxic and genotoxic effects of sublethal concentrations of aniline in *C. punctatus*. Symbolic elevation in time and dose dependent DNA damage was observed by comet assay as well as micronuclei assay revealing maximum damage after 96 h of exposure. After aniline exposure, scanning electron microscopy and ATR-FTIR studies showed anomalies in structure and alterations in biomolecules of RBCs of aniline exposed group as compared to control group respectively. Semi prep HPLC studies revealed bioaccumulation potential of aniline in higher concentration exposed group.

## Introduction

Each living thing on the planet earth values water as a vital resource. However, only 1% of water is actually available for human use, which is continuously being contaminated from various point and non-point sources of water pollution^1,2^. The scientific community is quite concerned about how diverse freshwater supplies are becoming increasingly contaminated. Businesses and sewage treatment facilities dump their wastewater straight into the environment. These industrial effluents contain contaminants that are infamous for their carcinogenicity, cytotoxicity, endocrine disruptive activity, ill effects on reproductive health, hormonal imbalance and numerous more detrimental consequences on aquatic life as well as on peoples^3^. Majority of industrial discharge contains manifold of organic contaminants. These are carbon based chemicals having immense utilization in different industrial processes. Some of the compounds are anisidine, aniline, nitro aniline, chlorinated anilines, pyridine, polycyclic aromatic hydrocarbons (PAHs) and polychlorinated biphenyls (PCBs) etc. These organic chemicals bind strongly to sediments and serve as a long-term source of contaminants in water bodies and biota long after the original source has been removed^4^.

Aniline (C_6_H_5_NH_2_) being an important intermediate in the organic and fine chemical industry is ubiquitously used worldwide. It is also called amino-benzene; phenylamine and is a common aromatic base. It is a parent molecule of the aromatic amine family and is one of the important building blocks for the manufacture of a variety of plastics, rubber additives, colourants, drugs, isocyanates, accelerators in rubber processing, dyes and pigments, photographic chemicals, fuel additives, antioxidants, corrosion inhibitors, pharmaceuticals and antiseptics^5^. Worldwide production of aniline in 2016 was estimated to be around 5.6 million tonnes. More than half of the world’s aniline output comes from North East Asia^6^. According to another source, statistics of aniline production in USA increased from production 4,49,000 tons in 1990 to 8,45,000 tonnes in 2019, similar increase in production was estimated in India during 2013–2019 which varied between 34,470 and 48,230 tonnes^7^. MDI (4,4-methylene diphenyl diisocyanate) which is used to produce polyurethane foam is the largest user of aniline. For the renovation of buildings and commercial constructions, polyurethane is used for insulating purposes. Hence with an increase in demand for construction industries, likewise there is increase in production of aniline. Numerous rubber-processing agents, including *N*-phenyl-2-naphthylamine, guanidines, aldehyde aniline condensates, thiazoles, and diphenylamine, use aniline as vulcanization accelerators and antioxidants. Furthermore, it plays an important roles in producing various pesticides (fungicides, bactericides, insecticides, etc.). It is utilized as precursors or intermediates for the production of more than 174 dyes besides varieties aniline derivatives were used in fabrication of more than 700 dyes^8^. Diverse anthropogenic sources can release aniline into the environment at any time while it is being produced, stored, transported, used, or disposed of, or while it is present in materials. Serious environmental pollution is created due to environmental-unfriendly nature of aniline^6,9^. Waste and discharge from several industries are uppermost source of occurrence of aniline in environment. Around 4–12% of azo dyes are lost to industrial effluents during dye production or textile colouration^10,11^. Aniline’s molecular structure becomes extremely stable when exposed to the environment. It readily attaches to colloidal organic matter, and changes the physical and chemical properties of water, sediment, and biological populations, resulting in the deterioration of water quality^1,12,13^. Due to aniline’s high toxicity and incalcitrant nature, pharmaceutical and dye industry wastewater containing it has been known to affect aquatic environments^14^.

Despite the fact that aniline has been regarded as a probable occupational carcinogen since the 1970s, there are few data on aniline exposure across all sectors and circumstances, where there is a high risk of exposure. In the European Union, aniline and its salts are no longer permitted to be used in any cosmetic items that are advertised for purchase or use. Nevertheless, it is used as predominant ingredient in tattoo inks which include more than 100 colourants and 100 additives, eventually causing direct human exposure to aniline. According to report by ECHA, the range of aniline content in tattoo inks is 5 to 61 mg/kg^15^.

Currently usage of aniline in several household products including fabrics, textiles and apparel, leather paper, plastic food packaging and storage, toys, mobile phones is progressively increasing but the studies revealing the occurrence, prevalence and toxic effects are very lesser^16–18^. So keeping in mind the present investigation has been undertaken to study the acute toxic effects of sublethal concentrations of aniline in fresh water fish *Channa punctatus* using haematology, genotoxicity, cytotoxicity, scanning electron microscopy and ATR-FTIR studies. Hematological study provide overview about different parameters viz, RBC count, WBC count, Hb level, Packed cell volume and mean corpuscular volume of blood tissue of different experimental groups. Comet assay and micronuclei assay were used to assess DNA damage in blood cells. Double staining method was used to assess cytotoxicity. Structural and functional alterations at molecular level is established using ATR-FTIR analysis. Fish have long been regarded as a useful model for assessing the toxic and mutagenic potential of various contaminants. Fish are occupants of all aquatic zones, they bioaccumulate contaminants from the environment, and respond to mutagens even at low concentrations, and provide early warning of changes in the environment brought on by pollution^19^. *Channa punctatus* is regarded as a superior test model species because it is extensively available and accessible all year long^20,21^. Moreover, easy maintenance in the aquaria and ease of non-invasive blood collection are the other important characteristics of *C. punctatus* fish making it suitable for laboratory use^22,23^. Also Bioaccumulation potential of the compound has been estimated.

## Results

### Median lethal concentration of aniline for fish Channa punctatus

After 96 h of exposure, there was no mortality up to 10 mg/l, and 100% mortality was recorded at 25 mg/l. The 96 h LC_50_ value of aniline was determined to be 16.78 mg/l for *Channa punctatus*. The selected sublethal concentrations were ¼ of LC_50_ and ½ LC_50_ that is 4.19 mg/l and 8.39 mg/l.

### Effect of aniline on haematological parameters

**Figure 1:**
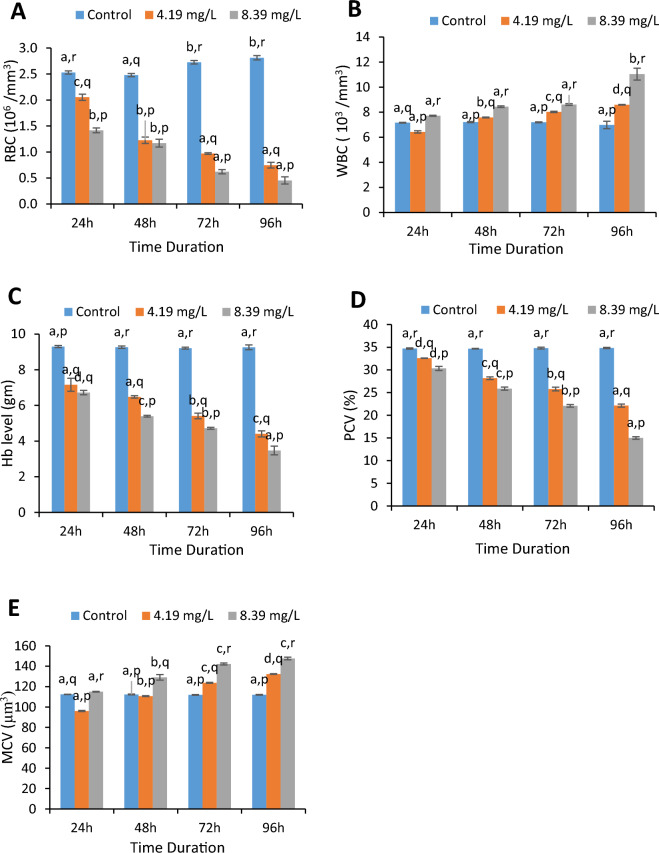
Represent (**A–E**) Red blood cell count (× 10^6^/mm^3^), White blood cell count (× 10^3^/mm^3^), Haemoglobin concentration (gram), Packed cell volume (PCV%) and mean corpuscular volume (MCV, μm^3^) in fish *C. punctatus* exposed to different concentrations of aniline for different hours. Different letters a, b, c, d signify the effect of duration of exposure and p, q, r signify the effect of treatment at the same time interval.

Up to 96 h, notable alterations in haematological parameters were perceived as represented in Fig. 1. After 96 h of exposure Hb content (g), RBC count (10^6^/mm^3^), and packed cell volume (%) explicated dose-dependent as well as time dependent significant declining trend as compared to control group. Highest decline in values was observed in 8.39 mg/l exposed group after 96 h of exposure. The value of Hb decreased from 9.26 ± 0.14 in control group to 3.47 ± 0.25 in higher concentration exposed group i.e. 8.39 mg/l, which was 62.52% lesser than the control value. Similarly, there was 56.95% decline in PCV value between control and higher concentration exposed group (8.39 mg/l) at 96 h of exposure. There was considerable decrease in RBC count from 2.81 ± 0.04 (control) to 0.45 ± 0.07 when exposed to 8.39 mg/l. Significant increase in WBC count (10^3^/mm^3^) was observed during different duration of exposure of aniline. Values of WBC count showed a significant hike at 96 h in higher concentration exposed group which was 1.58-folds as compared to the control group. Likewise, MCV value increased when going from control to aniline exposed groups in both time and dose dependent manner.

### Micronuclei assay

**Figure 2:**
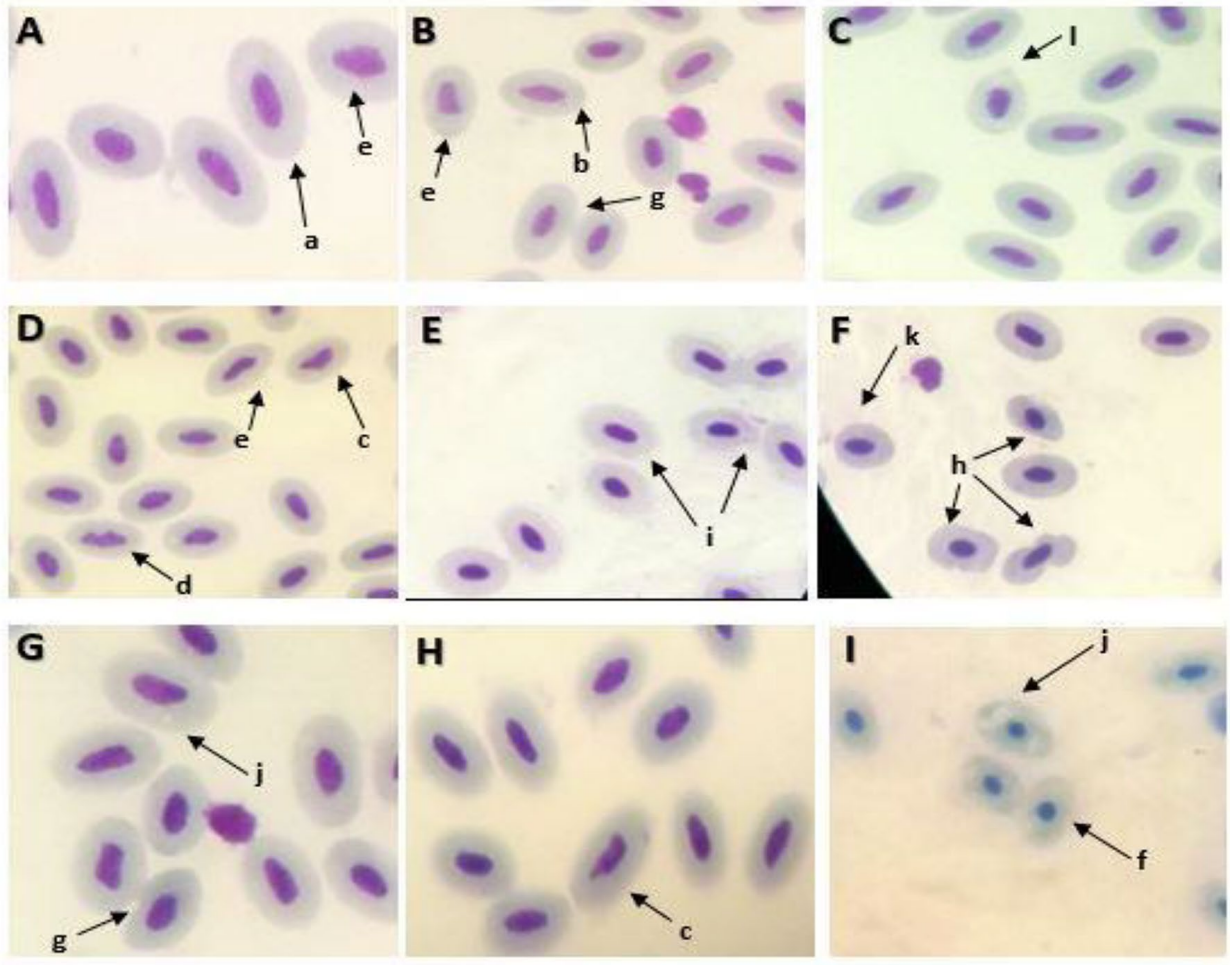
Photomicrograph showing different erythrocytic nuclear and cytoplasmic abnormalities in blood cells of *C. punctatus* after exposure to aniline. (**A**) Control group, (**B–E**) 4.19 mg/l aniline exposed group, (**F–I**) 8.39 mg/l aniline exposed group. (a) Normal erythrocytes, (b) micronucleated cell, (c) notched nucleus, (d) bilobed nucleus, (e) irregular shaped nucleus (f) pyknosis, (g) cytoplasmic bridge, (h) membrane breakage, (i) lysed cell, (j) vacuolated cytoplasm, (k) irregular shaped cell.

**Figure 3:**
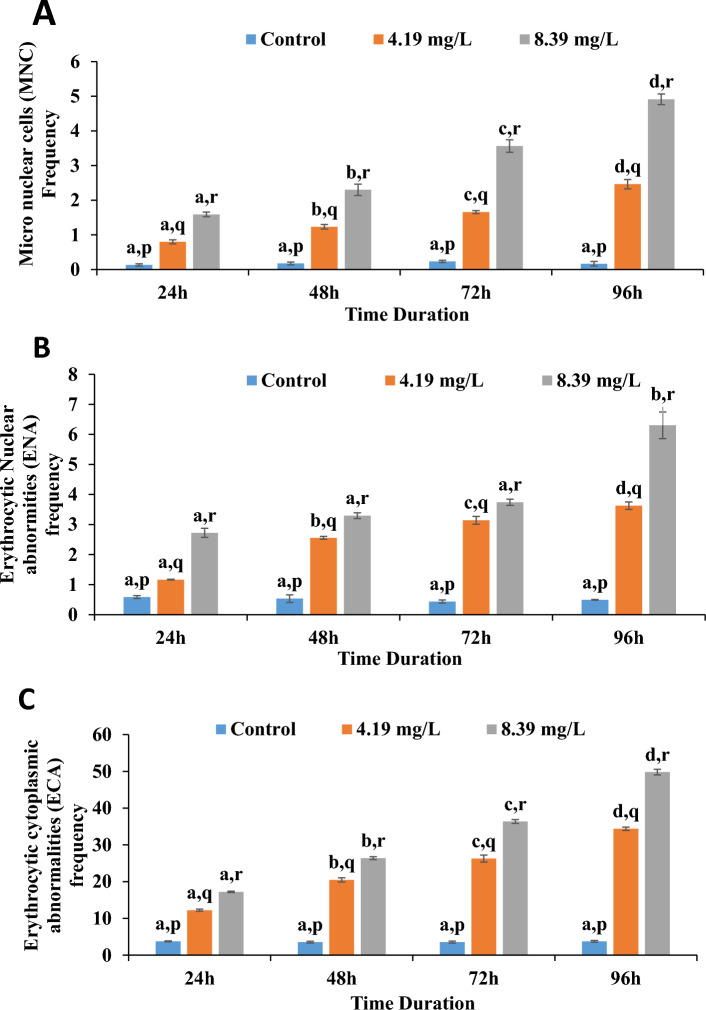
Effect of different concentrations of aniline on frequency of (**A**) micronucleated cell, (**B**) erythrocytic nuclear abnormities (ENA) and (**C**) erythrocytic cytoplasmic abnormalities (ECA) in blood cells of *C. punctatus* at different durations of exposure. Error bars represent standard errors (SE). Different letters a, b, c, d signify the effect of duration of exposure and p, q, r signify the effect of treatment at the same time interval.

Analysis of frequency of micronucleated, erythrocytic nuclear abnormities (ENA) and erythrocytic cytoplasmic abnormalities (ECA) in peripheral blood of control and aniline exposed group for different time period is given in Fig. 2. Mean MNC, ENA and ECA increased significantly in both the aniline exposed groups for 96 h (p < 0.05). Gradual amplification in frequency of abnormalities was observed with increase in time duration of aniline exposure. Utmost damage was observed on exposure to 8.39 mg/l at 96 h where MNC value raised from 0.17 ± 0.067 to 4.91 ± 0.154, ENA value raised from 0.49 ± 0.012 to 6.30 ± 0.443 and ECA value increased from 3.73 ± 0.237 to 49.80 ± 0.765 (Fig. 3). Erythrocytic nuclear abnormalities included notched, bulged, bilobed, pyknosis, irregular and elongated nucleated cells, whereas erythrocytic cytoplasmic abnormalities included cytoplasmic bridge, vacuolated, irregular, karyolized and bilobed cells.

### Comet assay

**Figure 4:**
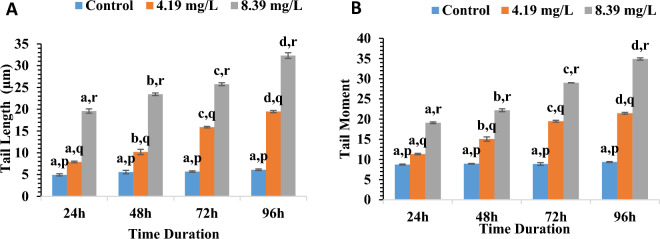
Effect of different concentrations of aniline on (**a**) tail length and (**b**) tail moment in blood at different durations of exposure. Error bars represent standard errors (SE). Different letters a, b, c, d signify the effect of duration of exposure and p, q, r signify the effect of treatment at the same time interval.

**Figure 5:**
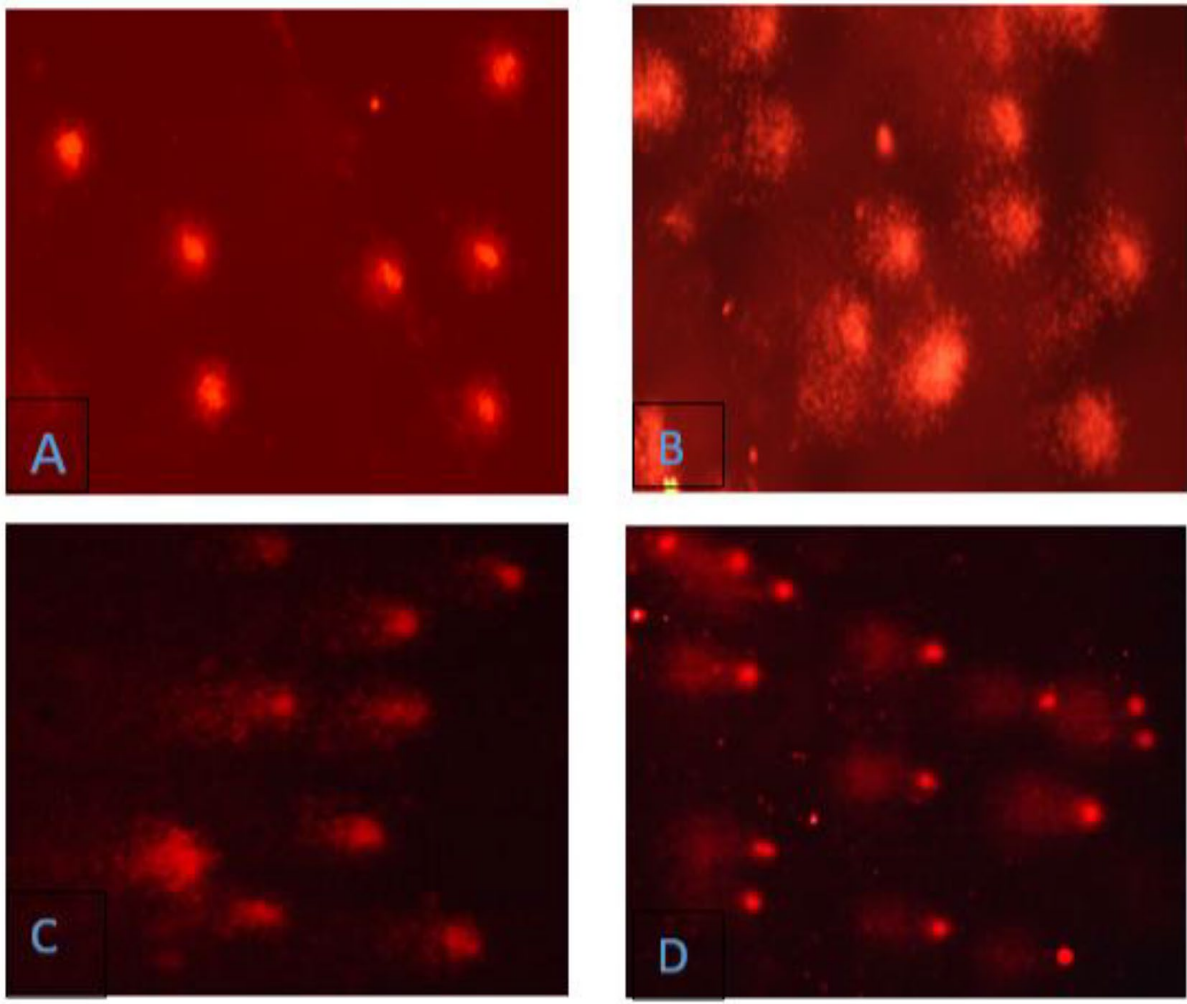
Photomicrographs showing different level of DNA damage in (**A**) Control group, (**B**) mild damage in 4.19 mg/l aniline exposed group, (**C,D**) severe damage in 8.39 mg/l aniline exposed group.

DNA damage after acute exposure was assessed using comet assay by investigating tail length and tail moment as parameters (Fig. 4). The impact of treatment with different sublethal concentrations of aniline on the tail length (TL) and tail moment (TM) in blood cells of *C. punctatus* is given in Fig. 5. Treatment with both the concentrations of aniline actuated significant change for both the parameters as compared to the control groups (Tukey’s test) shown in Fig. 5. Maximum damage was observed at 96 h of exposure with 8.39 mg/l where tail length increased 5.02-folds and tail moment increased 3.73 times in comparison with control group. The effect of duration of exposure was also found to be significant (One-way ANOVA, p < 0.05).

### Cell viability analysis by double staining method

**Figure 6:**
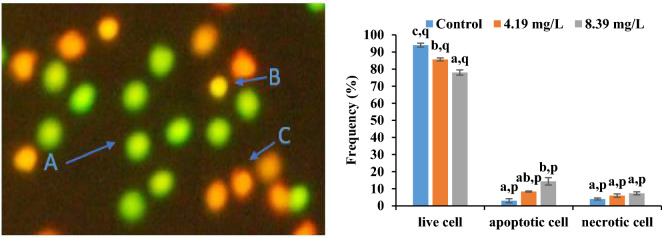
(**I**) photomicrograph showing effect of double staining on blood cells where (A) Live (viable) cells appeared as green (B) apoptotic cells appeared yellow (C) necrotic cells appeared as red in colour; (**II**) frequency of live cell, apoptotic cell and necrotic cell in *C. punctatus* after 96 h exposure to aniline. Error bars represent standard errors (SE). Different letters a, b, c, d signify the effect of duration of exposure and p, q signify the effect of treatment at the same time interval.

There was a significant concentration-dependent reduction in the frequency of viable cells after exposure to aniline for 96 h (Fig. 6). After 96 h of exposure, frequency of live cells declined by 8.86% at 4.19 mg/l and 17.2% at 8.39 mg/l respectively. After exposure for 96 h, necrotic and apoptotic cell frequencies, increased significantly in a concentration-dependent manner. Necrotic cell frequency increased by 1.5-fold and 1.8-fold at 4.19 mg/l and 8.39 mg/l respectively. Hike in apoptotic cell frequency was 2.78 times at lower concentration and 4.78 times at higher concentration exposure correspondingly.

### Scanning electron microscopy

**Figure 7:**
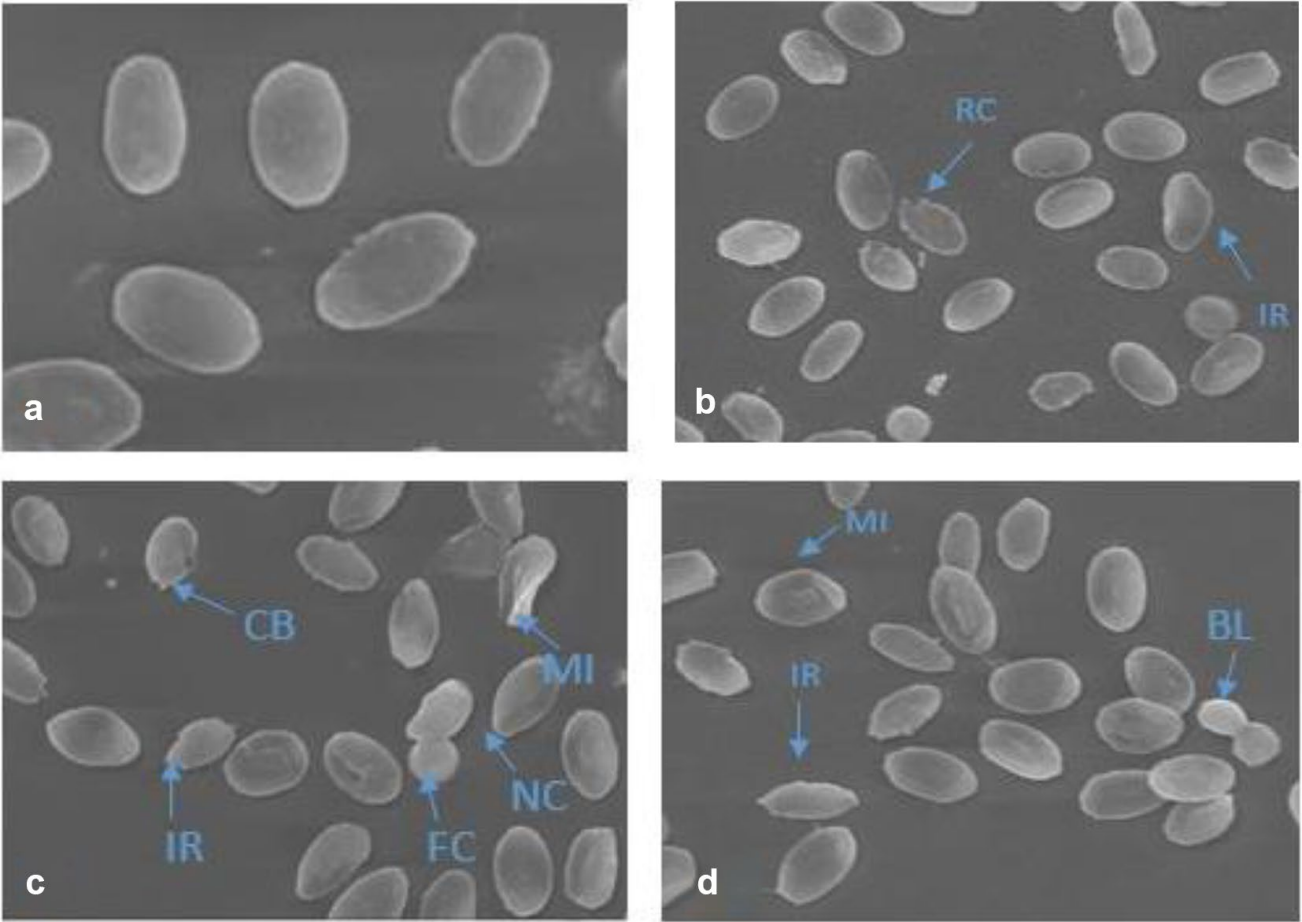
Photomicrograph of Scanning electron micrographs of erythrocytes of *Channa punctatus* after 96 h of exposure of aniline. (**a**) Control group, (**b**) 4.19 mg/l aniline exposed group (**c,d**) 8.39 mg/l aniline exposed group. *IR* irregular shaped cell, *NC* notched cell, *FC* fused cells, *MI* membrane internalization, *BL* bilobed cell, *CB* cytoplasmic bleb, *RC* ruptured cells.

Scanning electron microscopy showed different kinds of alterations in the structure of blood cells (Fig. 7). RBCs of aniline exposed groups showed uneven boundaries, irregular shaped cell, notched cell, fused cells, bilobed cell, cytoplasmic bleb, ruptured cells and membrane internalization.

### ATR-FTIR studies

**Figure 8:**
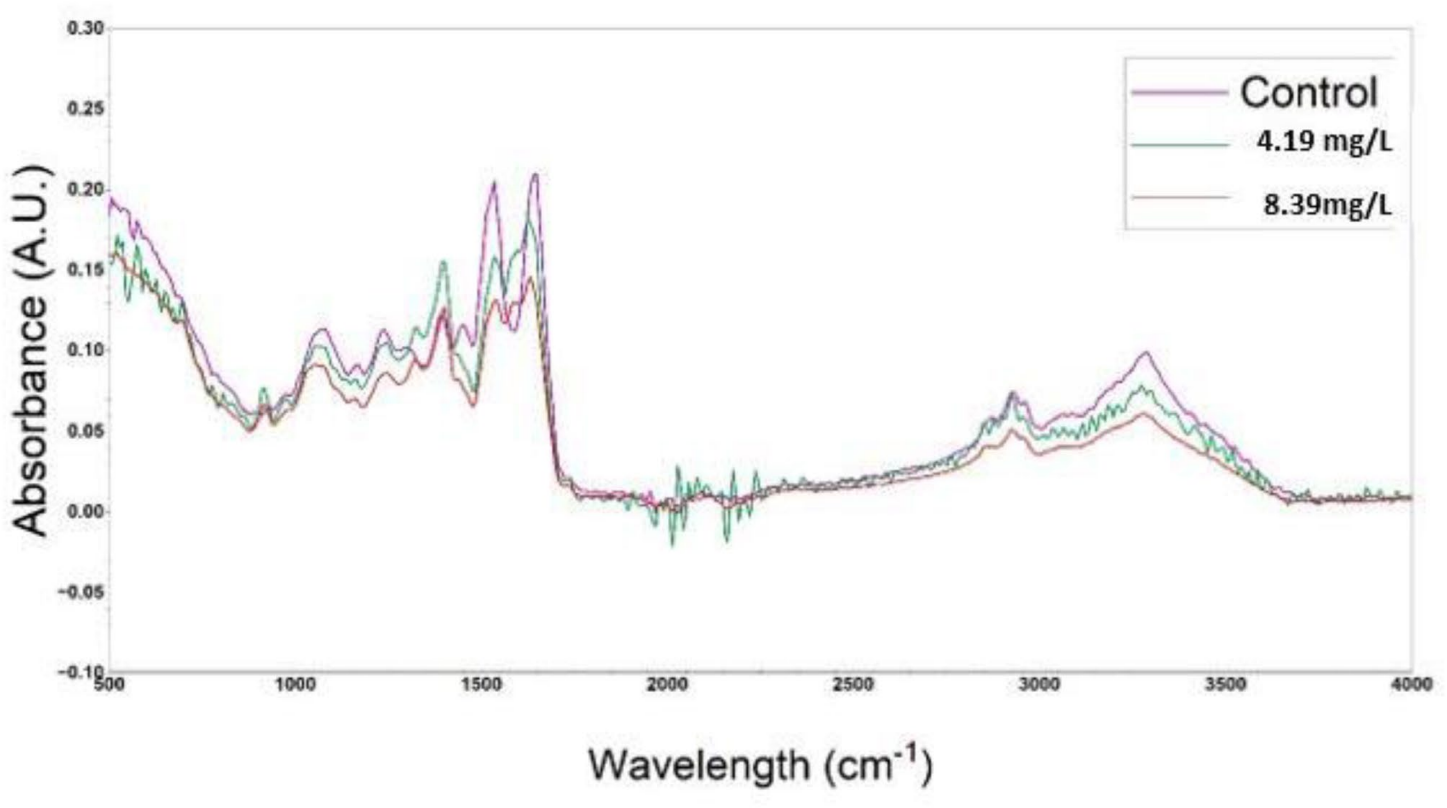
Represent mean FT-IR spectra of the control and different concentrations of aniline exposed RBCs of *C. punctatus* in the 500–4000 cm^−1^ region.

The present investigation also shows effect of aniline on different biomolecules of blood tissue of *C. punctatus* using ATR-FTIR technique. Alterations observed in peak absorption of infrared bands were assessed to obtain structural and molecular information about the particular sample (Fig. 8). Absorbance peak and band areas that correspond to different functional groups were assessed which highlights the alterations in structure and functioning of diverse biomolecules in control and aniline-exposed fish. Analysing the value for amide I (1654 cm^−1^) there is 25% and 37% decrease in absorbance and for amide II (1543 cm^−1^) there is 17% and 29.9% decrease in absorbance values in 4.19 mg/l and 8.39 mg/l exposed groups as compared to the control group. The bands observed at 1230 cm^−1^and 1081 cm^−1^ are due to asymmetric and symmetric modes of phosphodiester group present in nucleic acids respectively. The 24.2% and 21.2% reduction in absorption in highest concentration exposed group observed in these bands reveals symbolic genotoxic impact of aniline. At higher concentration exposure, 34% reduction in absorption value at 1454 cm^−1^ was observed which attributed to bending vibration of the CH_3_ in the lipids and proteins, similarly there was 20% decrease in absorption frequency at 1252 cm^−1^ which range for antisymmetric vibration of phosphodiester bond. There is reduction in absorption frequency in both the exposed group by 14.88 and 23.78% in the area of the lipid C=O stretching vibration (at 1744 cm^−1^) band which suggests an increased concentration of ester groups belonging to triglycerides.

### Bioaccumulation studies

The retention time was found to be 3.56 min and detector wavelength was 230 nm. The calibration plot for the assay of aniline was linear over the investigated range, i.e. 0.1–100 mg/l whereas the correlation coefficient was found to be 0.99 (p < 0.05). Samples for HPLC analysis were prepared from the blood cells of control and higher concentration of exposed group (8.39 mg/l). Sharp peak was obtained at 3.56 min in the aniline exposed group whereas no such elevation was obtained in samples from control group (Supplementary Fig. 1). Bioaccumulation of aniline was evaluated in blood of exposed group which was found to be 2.55 mg/l.

## Discussion

Aniline is a precursors or intermediate for the production of many dyes and personal care products and can be released into the surrounding through industrial effluents, accidental spills and their direct application to the soil. It can be exposed through dermal adsorption, inhalation, and ingestion. The European Chemicals Agency (ECHA) classifies aniline as acutely hazardous (Category 3), carcinogenic (Category 2), mutagenic (Category 2), skin sensitising (Category 1), and damaging to eyes (Category 1)^24–26^. Direct contact with it can cause eye damage that is irreversible, skin burns, and extreme discomfort. Fishes are the most reliable experimental model to study the effects of water-borne contaminants^19^. Acute toxicity to aniline has not been reported in *Channa punctatus* earlier. In the present study, 96 h LC_50_ value for aniline was calculated to be 16.78 mg/l. Earlier Abram and Sims^27^ calculated LC_50_ value for aniline in rainbow trout which is 8 mg/l whereas the LC_50_ for *Danio rerio* was evaluated to be 116 mg/l by Dom et al.^28^. The 96 h LC_50_ value of aniline was also evaluated for tilapia (69.38 mg/l) and for goldfish (187 mg/l)^29^. LC_50_ values determined for different animals were found to be varied due to the variations in tolerance levels among different organisms.

Aim of the present study was to evaluate the impact of acute toxicity of aniline via exposing *C. punctatus* to different sublethal concentrations of aniline for 96 h. Distinctive alterations were observed in different haematological parameters. There is significant increase in nuclear and cytoplasmic abnormalities in blood cells of test fish after different durations of exposure which was ascertained using micronuclei assay and scanning electron microscopy. Comet assay and cell viability assay implies the transformation at the DNA level due to the exposure of aniline, whereas ATR-FTIR studies validate alterations in structure and function at molecular level in different biological constituents (protein, lipid and nucleic acid). However, the bioaccumulation potential of aniline was also examined in blood cells of fish using HPLC analysis.

Blood haemoglobin (Hb) levels are routinely used to detect abnormalities and adverse indications of fish health^30,31^. Variation in the white blood cell count is considered as biochemical immunosuppressive indicators in fish^32,33^. In due course of experiment, significant changes were observed in the different parameters of the blood. It has been reported that different physiological parameters in fish were changed as a result of stress^19,34^. One of the most crucial indicators of physiological stress, which results from any variation that affects the homeostasis of fish, is blood parameters^35,36^. Erythrocytes count is considered as a quite stable index and fish species always maintain this index within the standard limit by compensating for different physiological techniques in stressful conditions^37,38^. In this study, there is notable decrease in RBC count, Hb level and packed cell volume after exposure to different sublethal concentrations of aniline which may cause disorders such as anaemia and erythropenia in fish. A similar decreased value of Hb was also reported in *C. punctatus*, when exposed to Bisphenol A for 96 h^19^. The significant reduction of Hb level and RBC count in the present study might be due to iron deficiency and structural alterations of haem, leading to decline haemoglobin synthesis. On the other hand, considerable increase in WBC counts may be caused by an increase in antibody synthesis, which enabled the organism to survive and recover when exposed to harmful contaminants^39,40^. This increase of WBC might be due to leukocytosis under chemical stress caused by aniline exposure, resulted direct stimulation of immunological resistance.

The most frequent indicator of toxicants present in water bodies is altered erythrocytes^41^. In the present study, different morphological (ECA and ENA) changes of erythrocytes were observed due to exposure of different sublethal concentrations of aniline. Changes in the erythrocyte structures may be due to the damage of the cell membrane, ion-permeability, and absorption of toxic substance by cells^42^. The exposure of aniline affects the integrity of DNA which consequently influence the structural and functional aspect of nucleus and cytoplasm of cells which was revealed by *Micronuclei* assay (erythrocytic nuclear and cytoplasmic abnormalities), comet assay and scanning electron microscopy. In current research, both duration and dose dependent increase was observed in micronucleated, erythrocytic nuclear and cytoplasmic cell frequency in aniline exposed groups. Similar study by Srivastava^43^, revealed erythrocyte morphological alterations in freshwater fish *Channa punctatus* under exposure of nigrosine black. Different studies revealed that the nucleus of fish erythrocyte plays a vital role in all metabolic and genetic activity^30,38,44^.

Nuclear abnormalities such as nuclear buds, elongated nuclei, and lobed nuclei may be the result of harmful consequences caused by clastogenic contaminants. Gene amplification causes all of these modifications, which obstruct the chromosomal connection and cause the formation of notched, budded, binucleated, and other deformed nuclei^22,45,46^. The clastogenic pollutants increase the mutation frequency in DNA, which leads to different types of nuclear and cellular abnormalities which can be easily observed with the help of MN assay^47^. The comet assay or single-cell gel electrophoresis (SCGE) technique is a method for measuring DNA strand breaks in individual cells and is used to evaluate genotoxicity, DNA repair, and genome instability. Due to its ability to detect very low-level oxidative damage caused in DNA, the SCGE is a well-established assay for the assessment and estimation of DNA damage both in vitro and in vivo*,* at the individual cell level^45^. Comet test is said to be one of the most advantageous tool for evaluating genotoxicity since it is a simple and reliable indicator for determining DNA damage. Up to 96 h after exposure, a significant dose- and time-dependent rise in TL and TM was seen. The interaction of aniline with DNA that results in DNA strand breakage, the production of adducts and crosslinks, faulty transcription, cellular malfunction, and a host of other disorders in the organism may be the cause of this substance’s genotoxic potential.

Aniline after entering the body might disturb the antioxidant system of the fish and leads to the production of reactive oxygen species (ROS), which may interact with DNA and other cellular biomolecules, resulting in damaging them. Role played by ROS in cell survival, apoptosis, and death has been depicted by Franklin^48^. The cyto-genotoxic effects of the selected concentrations of aniline were clearly visible from a concentration-dependent increase in the frequency of necrotic and apoptotic cells and the elevation of MN, nucleo-cytoplasmic abnormalities and comet parameters. Martin et al.^49^ suggested that the main reason behind apoptosis caused by a xenobiotic is the activation of endogenous nucleases and the formation of oligo-nucleosomal fragments of DNA. The fragmentation of DNA is directly associated with an increase in the frequency of apoptotic cells as is clear from the concentration dependent increase in nuclear fragmentation and cell lysis^50^. When defence system of a cell is unable to repair severe DNA damage, the damaged cell undergoes necroptosis or necrosis. Erythrocytes from *C. punctatus* showed a variety of abnormalities in SEM examinations, including uneven form, notched, elongated cells, and cytoplasmic blebs. Various researchers observed different abnormalities in erythrocytes of fish exposed to different xenobiotics^19,51–53^.

FTIR spectroscopy monitors the vibrational modes of functional groups within biomolecules and enables a correlation between chemical information and histological structure^54^. The spectral region of 1800–1500 cm^−1^ (amide I and amide II) contains mainly information on the protein content and its secondary structure. In general, α-helical structures have a band peak at wavenumbers 1650–1658 cm^−1^; β-sheet structures tend to have bands between 1620 and 1640 cm^−1^and between 1670 and 1695 cm^−1^; random coil structures occur at around 1644 cm^−1^^55–57^. Literature studies reveal that the assessment of protein secondary structure could be made on the based on the analysis of the Amide I band and amide II band^58,59^. In the current study, there was significant differences at the frequencies of 1543 cm^−1^ and 1654 cm^−1^, corresponding to the absorption bands of amide II and I, respectively. This hypothetically may indicate conformational changes in serum proteins^60,61^.

The changes of functional groups like PO_2_, C–O, and C–C, which are found in proteins, lipids, nucleic acids, and carbohydrates, are referred in the zone of 1300–800 cm^−1^^57^. The relatively strong bands at 1230 and 1080 cm^−1^ are mainly due to asymmetric and symmetric stretching modes, respectively, of phosphodiester groups in nucleic acids rather than in phospholipids. The band observed at 1744 cm^−1^ is assigned to C=O stretching vibration of ester groups in triacylglycerol. Decrease in absorption value at 1744 cm^−1^ indicated that the content of lipids decreases in the treatments groups. The major consequences could be ionization and excitation of the triacylglycerol content of lipid bond disruption between the fatty acid and glycerol moieties which caused formation of the dominant triacylglycerol radical. Excited triacylglycerols can also undergo a wide variety of reaction which could affect body homeostasis^62,63^. Using IR spectroscopy, one can analyze the blood serum’s entire biochemical signature (including proteins, lipids, nucleic acids, and carbohydrates) instead of focusing on one specific protein as a biomarker^64^. The frequency of oscillations in the structure of DNA and RNA can be utilised as a stress indicator in toxin exposed groups, while analysing the IR spectra of blood sera^65,66^.

The bioaccumulation of xenobiotics is thought to increase ROS, causing an imbalance between the fish’s internal stress-compensation systems and the development of oxidative stress. Accumulation of aniline in higher concentration exposed group ½ LC_50_ (8.39 mg/l) was investigated after duration of 96 h. The retention time for aniline at 230 nm was 3.56 min. After evaluation of blood after 96 h, 2.55 mg/l concentration of aniline was observed in blood sera of *C. punctatus.* Earlier studies by Hardy et al.^67^ revealed bioaccumulation potential of aniline and phenol in freshwater phytoplankter S*cenedesmus quadricauda.* Aniline has a greater potential for bioconcentration with bioconcentration factor (BCF) 91 than phenol with bioconcentration factor 3.5. Morales Trejo et al.^68^ performed a similar study, which revealed the presence of clenbuterol with the help of HPLC and considered quantification process as one of the application of HPLC. Currens^69^ estimated Bioconcentration Factors for Organic Chemicals Using High Performance Liquid Chromatography (HPLC) and revealed the higher BCF for aniline and its derivative components. The current study highlighted different outcomes of aniline exposure on blood tissue of treated sample. Alterations in different hematological parameters, DNA damage, elevation in necrotic cells and modification in the ATR-FTIR values of treated group represent toxicant response of aniline concentrations on test model *C. punctatus.* It is one of the significant and harmful health risk to both terrestrial and aquatic species due to its widespread usage and ongoing industrial discharge.

## Materials and methods

### Experimental fish and test chemical

Healthy and active freshwater food fish *Channa punctatus* (Bloch; family; Channidae, Order; perciformes) were procured from local fish market, Amritsar. The average weight and height of fish were 22 ± 2 g and 14 ± 2 cm respectively. 0.1% potassium permanganate (KMnO_4_) was used as a preclusive measure to avoid any kind of dermal infection. Fish were reared in static conditions in glass aquarium of 100 l water capacity, under a natural photo regimen (14/10 h, light/dark) for a minimum period of 15 days. During the acclimatisation period fish were fed with local fish feed once a day and boiled egg yolk is provided to the fish twice a week. To reduce the ammonium content and maintain the water quality of tank, uneaten food and faecal matters were removed with the help of a scoop net and siphon. Furthermore, aquarium water was exchanged with freshwater every alternative day. For the purpose of experiment, height and weight of healthy fish were also noted. All the experiment protocols have been approved by the Institutional Ethics Committee of Guru Nanak Dev University, Amritsar (letter no. 226/CPCSEA/2021/022).

Aniline was purchased from Sigma Aldrich, India (CAS NO 62-53-3 and purity 99.5%). The substance had a musty, fishy aroma and was a yellow to brownish greasy liquid. Since aniline dissolves in water at rate of about 2 g/l, no stock solution was made for the experiment and aniline was simply dissolved in aquarium water to maintain the various test concentrations.

### Water parameters

The physicochemical properties of test water were analysed using water analysis kit. The values of different water parameters analysed during the experiment were ranged as follow: temperature 24–26 °C, pH 7.4–7.7, total dissolved solids (TDS) 0.34–0.37 g/l, dissolved oxygen (DO) 4.7–4.9 mg/l, total conductivity 527–532 ms/cm and hardness 229–231 mg/l respectively.

### Estimation of 96 h LC_50_ for aniline

After acclimatisation, fish were randomly selected and batches of 10 fish each were transferred to separate aquatic tanks where static bioassays were conducted to estimate median lethal concentration (LC_50_) of test chemical for 96 h. Toxicity range for aniline was found to be in between 10 and 25 mg/l on the basis of percentage mortality. For evaluation of the 96 h LC_50_ value, in static renewal system test water was changed every day to sustain the dissolved oxygen content and removal of excretory waste. In order to keep the chosen concentration of aniline in the test environment, it was also added again each day to the test water. To find out the lethal concentration which kills 50% of the test animal within 96 h, fish were exposed to different concentrations viz., 10, 12, 14, 16, 18, 21, 24 and 25 mg/l of aniline. The percent mortality corresponding to different concentration values were then computed at a p < 0.05 level of significance using probit analysis^70^.

### Experimental design

After determining the median lethal concentration for aniline that is LC_50,_ two sublethal concentrations were selected to evaluate the impact of acute exposure of aniline on test animal. The selected sublethal concentrations were ¼ of LC_50_ (4.19 mg/l) and ½ LC_50_ (8.39 mg/l) and for each parameter experiment was repeated thrice. Blood samples were collected by puncturing the cardiac region using sterile needle after different duration of acute exposure. Three fish per duration were selected for experiment. For genotoxicity analysis, samples were collected after interval of 24, 48, 72, 96 h and were immediately processed for micronuclei assay, comet assay and for evaluation of different haematological parameters samples were processed. Cytotoxic effects of toxicant exposure were assessed by performing double staining cell viability assay. After 96 h of exposure to aniline structural alteration in blood cells were observed by scanning electron microscopy. ATR-FTIR were performed after collecting samples from 96 h exposed fish which provide an overview about the variation at molecular level in blood tissue. Accumulation potential of aniline in fish group exposed to highest concentration (½ LC_50_) was evaluated using semi prep HPLC after 96 h of acute exposure.

### Haematological studies

Blood was drawn out by puncturing the heart of fish using sterile needle in triplicate after 24, 48, 72, 96 h and collected in EDTA coated Eppendorf tubes. Blood samples were analysed using auto hematoanalyzier—Horiba ABX Micros60^71^. Different blood parameters like Red blood cells count (RBC count), white blood cells count (WBC count), haemoglobin content (HB), packed cell volume (PCV, %) and mean corpuscular volume (MCV, μm^3^) were calculated.

### Micronuclei assay

Peripheral blood was collected and smear was prepared on pre-cleaned microscopic slides after 24, 48, 72 and 96 h. For the fixation, smeared slides were placed in methanol for 10 min and then left to air dry at room temperature. For staining of slides, 5% Giemsa stain solution was prepared. Slides were placed in staining solution for 15–20 min. For each group (control and sublethal concentrations) 1000 cells each were examined for Micronucleated Cells (MNC), Erythrocytic Nuclear Abnormities (ENA) and Erythrocytic Cytoplasmic Abnormalities (ECA) under binocular microscope OLYMPUS CX2^72^.

### Single-cell gel electrophoresis (comet assay)

Alkaline version of Comet assay was performed using blood samples by using methodology of Tice^73^ with slight modifications. By puncturing the heart, 10 µl of blood is immediately diluted in 1 ml of phosphate buffer saline and stirred vigorously. Slides coated with 1% of normal melting point agarose (NMPA) and then were incubated at 37 °C for overnight in incubator. 0.5% of low melting point agarose (LMPA) mixed with 20 µl of blood was added to the coated slides and after loading the samples, slides were kept at 4 °C for 15–20 min. Third layer of 0.5% LMPA was poured on the slides and placed in refrigerator at 4 °C for another 15 min. Slides were then placed in lysis buffer for about 3 h in the refrigerator and then slides were placed in electrophoretic unit which contain electrophoretic buffer for a run of 20 min at 300 mA and 24 V. After the assigned time in electrophoretic unit, the slides were neutralized using neutralizing buffer for 15 min. Then slides were kept for drying overnight. Ethidium bromide was used to stain the slides before they were kept for observation under Nikon ECLIPSE E200 (Tokyo, Japan) fluorescent microscope with × 40 magnification, excitation filter of 515–560 nm. CASP LAB image analysis software was used for examining the comets**.**

### Cell viability analysis by double staining method

This method of detecting apoptosis is based on the loss of plasma membrane integrity as cells die^74^. Stock solution of both the dyes 100 µg/ml ethidium bromide and 100 µg/ml acridine orange was prepared in Phosphate Buffer Saline (PBS). Blood samples from control and sublethal concentration groups were collected after 96 h of exposure. 5 µl of blood and 10 µl of dye mixture (equal volume of both the ethidium bromide and acridine orange) were taken on slide, covered with coverslip and immediately examined using Nikon ECLIPSE E200 (Tokyo, Japan) fluorescent microscope at × 40 magnification. Cells identified as Live (viable) cells appeared green, apoptotic cells appeared yellow and necrotic cells appeared red in colour^75^. During the scoring, total of 100 cells were scored from each group with three replicates each.

### Scanning electron microscopy

Blood samples was collected from control and treated fish. Three to four drops of blood were immediately fixed in 2.5% glutaraldehyde prepared in 0.1 M phosphate buffer (pH 7.4) for 2–3 h and the sample was centrifuged at 1500 rpm for 5 min. Supernatant was discarded and the pellet was washed with phosphate buffer 2–3 times. The pellet was suspended in a small volume of distilled water and dehydrated with increasing concentrations of ethanol. On a circular coverslip of 10 mm, a small drop of suspension was applied and the sample was air dried. The sample was then sputter coated with gold and examined under a scanning electron microscope at an accelerating voltage of 15–20 kV.

### ATR-FTIR studies

Blood sample collected after 96 h from control as well as both sublethal concentration groups, was washed with phosphate buffer saline and freeze dried for next 24 h. The sample was then lyophilized and sublimated to powdered form to remove the water content. Freeze dried tissue sample powder (0.1 mg) was placed directly on the surface of a Cary 630 FTIR Spectrometer (Agilent Technologies) coupled with a diamond attenuated total reflectance (ATR) accessory. The samples were scanned in triplicate at room temperature in the spectral range of 3300–900 cm^−1^.

### Bioaccumulation studies

For present investigation, Shimadzu semi prep HPLC system was used, which is equipped with fraction collector and UV/Vis detector. C-18 analytical column was used and the mobile phase were in ratio of acetonitrile (80): double distilled water (20) with flow rate of 1 ml/min and temperature was maintained at 30 °C. All the solvent used during the experiment were filtered using filtration assembly whereas samples and standard solutions were filtered through 0.45 µm millipore syringe filters. The detection wavelength for aniline was set at 230 nm. Stock solution was prepared using HPLC grade chemicals from which working standard solutions were prepared by gradual dilution with mobile phase. Standard curve was plotted using peak area of known concentration and quantification of unknown concentration of aniline was evaluated. For evaluation of bioaccumulation potential of aniline in blood plasma, blood samples were collected from higher concentration group (8.39 mg/l) and control group after 96 h of exposure. Samples were centrifuged at 3000 rpm for 15 min at 4 °C. Vortexed for 2 min and then kept overnight at room temperature. Crude sample was mixed with equal volume of acetonitrile and filtered via millipore 0.45 µm syringe filter. The resultant was loaded into column with 10 µl of injection volume. Peak identification was made by comparing the retention time of samples with that of standard solutions. Three replicates from plasma of each group were evaluated. The peak area versus concentration data were used for further analysis as subjected by Bhandari and Kaur^76^ and Mehra and Chadha^77^.

### Statistical analysis

Statistical analysis was performed using a software program (SPSS 20.0 for windows). Data was expressed as mean value with standard error (mean ± SE). Statistical significance was examined on the basis of a comparison of averages using a one-way analysis of variance (ANOVA). In order to evaluate statistically significant differences between the tested samples, a multiple comparison test—the Tukey test (post-hoc) was used. The differences were considered to be statistically significant when p < 0.05.

### Ethical approval

All the experiment protocols have been approved by the Institutional Ethics Committee of Guru Nanak Dev University, Amritsar (Letter No. 226/CPCSEA/2021/022).

## Conclusion

The present study revealed the outcome of exposure of aniline at different sublethal concentrations in freshwater fish. Results emphasise about the haematotoxic, cytotoxic and genotoxic potential of aniline at concentrations i.e. 4.19 mg/l and 8.39 mg/l. Exposure of aniline for acute duration affects different structural and functional aspects of blood tissue of treated animal. This study could layout foundation to revaluate the pertinent policies regarding the usage, management and release of such toxic compound in surrounding. Studies revealing the alteration in different organs and modification in metabolism of aniline exposed organism need to be done. Strict policies should be made to stop direct discharge of aniline in waterbodies. Hence health status could be maintained for aquatic organisms as well as for waterbodies.

### Supplementary Information

Supplementary Figure 1.
