# Supplementary material for: Evaluation of haematological, genotoxic, cytotoxic and ATR-FTIR alterations in blood cells of fish Channa punctatus after acute exposure of aniline
Source: Sci Rep. 2023 Nov 25;13:20757. doi: 10.1038/s41598-023-48151-z (PMC10676417; doi:10.1038/s41598-023-48151-z)
Supplement: Supplementary file 1 — Supplementary Figure 1. [file 41598_2023_48151_MOESM1_ESM.docx]

Supplementary data:

2.8. *Bioaccumulation studies*





**Supplementary figure 1:** Graph showing peak structure of aniline in standard, control and aniline treated samples of *C. punctatus.*
